# Congestion of the Uterus, Its Causes and Treatment

**Published:** 1897-04

**Authors:** John Thompson Graham

**Affiliations:** Wytheville, Virginia


### Southern Medical Record.

A MONTHLY JOURNAL OF MEDICINE AND SURGERY.

VOL. XXVII. ATLANTA, GA., APRIL, 1897. No. 3

### Original Articles.

### CONGESTION OF THE UTERUS, ITS CAUSES AND TREATMENT.

BY JOHN THOMPSON GRAHAM, M. D., WYTHEVILLE, VIRGINIA.

For the last ten or twenty years the gynecologist has taken from the practice of the general practitioner the majority of the diseases of the female pelvic organs, and at one time it looked as if every woman in the country would soon have her uterus, tubes, and ovaries placed in pickling jars, to be stowed away in the office of some enterprising abdominal surgeon, or on the shelves of the laboratories of our numerous hospitals. But the tide of surgical gynecology which has swept over the country has, to some extent, changed, and the general practitioner finds that he has yet a field for non-surgical work in the treatment of diseases of the female generative organs. Surgery of the pelvic and abdominal organs is just as legitimate as it is in other parts of the body when the pathological conditions of these organs demand it; but that it has been resorted to in too many cases, and without a proper trial of non-surgical treatment, is clearly proven by the records of the past ten years of surgical gynecology. It is equally true that many diseases of the uterus and its appendages can be prevented, and many others cured, by conservative treatment if their causes are studied in the light of the pathology of inflammation and its rational treatment.

It is the purpose of this paper to consider some of the diseases of the uterus that may be treated successfully by the general practitioner, and as a term to include these diseases, I use the word congestion, as it is a condition always to be found from first to last in nearly all uterine affections.

By congestion of the uterus is meant that condition of the organ in which it is found to contain too much blood, and is therefore increased in size and weight, and often tender and painful.

Congestion here as in other parts of the body, may be either passive or active, and its causes are numerous. First, it may be asserted as a general proposition that anything that interferes with the circulation of the blood through the pelvic organs will, sooner or later, produce congestion of those organs. Under this head may be mentioned the faulty positions of the body assumed by women who lead a sedentary life, such as the cramped position required in the use of the sewing machine, and the very ungraceful position acquired by those who sit for hours with crossed legs and body bent forward in working fancy articles, such as embroidery, and other decorative arts. In this position the blood can reach the lower half of the body through the arteries, which are elastic tubes and are not easily compressed, but it can not get back through the veins whose walls are non-elastic but are compressed at every angle of the body. The result is that too much blood collects in the pelvic cavity, the veins of the pelvic organs being surrounded by elastic cellular tissues are over-distended and we have a passive congestion. Improper clothing is another great adjuvant to passive congestion of the uterus. Beginning from the body we have the drawers, the corset, corset cover, and from one to three skirts varying with the season, then the dress skirt, which fashion has decreed shall be at least seven yards in width, all compressing and hanging from the waist.

Congestion is also produced by constipation and allowing the bladder to be over distended. This is especially common in women who take very little out-door exercise, and in all women during the winter season who have not the convenience of the modern well-heated water closet.

Active congestion of the uterus may be produced by anything that drives the blood suddenly or with too great force to the pelvic organs. One of the most common causes of active congestion is the sudden chilling of the surface of the body when overheated from exercise, and especially during menstruation. This is one of the most prolific causes of uterine diseases in every class of society; the servant and washer-woman in performing their duties often have their bodies overheated while their feet are damp and the lower limbs are chilled for lack of sufficient clothing. The women of the upper circles suffer the same results by going out into the cold air from overheated buildings such as churches, theaters, and the ball-room. Another cause which is getting to be more common among the higher classes, is the prevention of conception by any means whatsoever; but especially to be condemned is the use of cold vaginal douches just after coition, when the generative organs are in a state of excitement. If a stream of cold water is thrown into the vagina, the vessels are suddenly contracted but soon a reaction occurs, and when often repeated, congestion with its numerous results will exist.

Attempts at abortion, successful or unsuccessful, will in nearly every instance cause severe congestion of the uterus and often lead on to severe and dangerous inflammations, such as cellulitis and peritonitis. Subinvolution which is often a chronic congestion is most frequently caused by too rapid child-bearing, or some imprudence after labor.

Incomplete development of the uterus and ovaries during the first few years of puberty is responsible for much trouble in after life. This is especially observed in ambitious girls who are overworked in mind at the expense of the body's development. Boys and girls are not treated alike in this respect. They engage in the same exercises while children, but when they are separated and sent to different schools their physical development widely differs. Our female colleges are sadly lacking in a system of exercises for the proper development of the physical structure, and just as soon as a girl is old enough to send off to college the decrees of fashion impose a bondage upon her actions. "The decorous girl must abandon her romps and games, and be content to confine herself in corsets that embrace the waist with a tighter and steadier grip than any lover's arm, and skirts that weight the hips with more than maternal burdens;" she must don tight shoes and assume the regulation walk.

It is just at this period that muscular development should be most looked after, and the aim should be to eradicate any special defect or weaknesses, and then to create and maintain a symmetry of all the parts, gradually increasing the strength and bodily vigor up to maturity, in order that we may have a sound mind in a sound body.

If we take time to compare the girls of to-day with those of the past we must realize that there is something wrong in our present system of education. The ideals beheld in pictures and statues of the past were models of health and beauty; they show a well developed and shapely arm and shoulder, a high chest and vigorous body, and a firm and erect carriage. Instead of viewing this galaxy of beautiful parts to-day, a vision of pipe-stem arms, scrawny necks, angular shoulders, flat chests, narrow backs, a stooping carriage and a weak and mincing walk recalls us to the realities of the present.

If we still further compare the brilliant eye, the damask cheek, and the luxuriant form of the robust English damsel with the vacant gaze, pallid features, attenuated forms of the easily fatigued, languid girls, the products of our modern American homes and customs, the contrast is still more strik ing. It shows that our English cousins are developing into strong and healthy women, while our American daughters are being reared as hot-house plants and are unable to perform the duties required of them, and are often wrecked in health, of both mind and body, early in life to spend the remainder of their days as miserable invalids.

Another very common cause of congestion of the uterus is some one of the faulty positions common to the organ: no matter whether it be retroversion, anteversion, or lateral version. The circulation is interfered with, congestion will be produced and finally prolapsus will be the result.

### TREATMENT.

The treatment should be both constitutional and local.

First, constitutional and general treatment.

It must not be forgotten that a woman has some organs outside of the pelvis that are liable to get out of good working order, and it is useless to try to cure a congested uterus or any of its various complications when the patient is at the same time suffering from an inactive or torpid liver, constipation and malassimilation. The excretory organs must also be looked after, especially the skin and kidneys—both of which must perform their natural functions before health can be restored. Tonics and fresh air are usually needed in every case. Some pleasant exercise should be engaged in, and for those who have no definite aim in life an effort should be made to get them interested in some light work or study in which they can employ their minds, and in this way have less time for morbid introspection.

Faulty clothing must be replaced by such as will allow the lungs and muscles to act to their full capacity uninterrupted by the dictates of fashion

### LOCAL TREATMENT.

The purpose of local treatment is to relieve the congested uterus of its excess of blood and thus reduce its size and weight in order that when restored to its natural position it will remain in place.

The action of local treatment depends upon the law of osmosis and capillary attraction.

No matter what is applied to the inflamed organ, whether glycerine or other applications, the principle of their action is the same, with the exception of hot water and iodine. Hot water relieves congestion by means of its tonic effect upon the blood vessels, causing them to contract and therefore they will contain less blood, but as it is used by the majority of women while in a sitting position its action is very imperfect and unsatisfactory. To get the full benefit to be derived from hot water the patient must be placed on her back and the douche given by a physician or trained nurse, and not less than one gallon of water should ever be used.

Iodine is beneficial in chronic congestion on account of its alterative effect, and when combined with carbolic acid, known as Battey's Iodized Phenol, it is an excellent application in old cases of cervical endometritis. In these cases the cervical canal is nearly always found to contain a tough mucus which is very hard to remove.

I have found peroxide of hydrogen to be the best agent to dissolve this mucus. It is applied on a cotton-wrapped probe in fifteen volume strength.

Glycerine, the old, time-honored agent in treating congestion of the uterus, is still the one most commonly used, either alone or combined with other agents; besides its endosmotic action it has a strong affinity for water which it takes up very rapidly from any inflamed surface to which it may be applied. It is usually applied to the uterus on sponges, cotton, or wool; the latter being the best agent to use as a tampon, because it is elastic, and does not prevent drainage.

Some of the discharge which is produced by the application of tampons to the uterus is perhaps due to the effort of nature to get rid of any foreign body. Whenever a foreign body of any kind comes in contact with a mucus membrane an increased flow of mucus and serum is produced in an endeavor to wash away the foreign substance. This is observed when a cinder is blown into the eye, a grain or bean is put up into the nose, or a crumb of bread gets lodged in the throat; and I believe to some extent the same law will apply to tampons in the vagina.

Any faulty position of the uterus must be corrected before the congestion can be relieved, and often the much-abused pessary will be needed, especially in cases of long standing in which we find the natural supports utterly worn out and relaxed; yet we must remember that the pessary is only an artificial support which should be relied on only while the natural tone is being restored to the uterine ligaments by local and constitutional treatment.

In the treatment of congestion in the vagina I have found that glycerine suppositories, with which I have lately combined thymol, alum, and benzoate of soda, are very effective in relieving the inflammation; and they can be used without an examination with the speculum, or the patient can be taught to apply them herself.

